# Influence of ecological factors on the presence of a triatomine species associated with the arboreal habitat of a host of *Trypanosoma cruzi*

**DOI:** 10.1186/s13071-018-3138-4

**Published:** 2018-10-29

**Authors:** Sofía Ocaña-Mayorga, Simón E. Lobos, Verónica Crespo-Pérez, Anita G. Villacís, C. Miguel Pinto, Mario J. Grijalva

**Affiliations:** 1 Centro de Investigación para la Salud en América Latina (CISeAL), Escuela de Ciencias Biológicas, Facultad de Ciencias Exactas y Naturales, Pontificia Universidad Católica del Ecuador, Calle San Pedro y Pamba Hacienda, 170530 Nayón, Ecuador; 2 Museo de Zoología, Escuela de Ciencias Biológicas, Facultad de Ciencias Exactas y Naturales, Pontificia Universidad Católica del Ecuador, Av. 12 de octubre 1076 y Roca, 170525 Quito, Ecuador; 3 Laboratorio de Entomología, Escuela de Ciencias Biológicas, Facultad de Ciencias Exactas y Naturales, Pontificia Universidad Católica del Ecuador, Av. 12 de octubre 1076 y Roca, 170525 Quito, Ecuador; 4 Instituto de Ciencias Biológicas, Escuela Politécnica Nacional, Ladrón de Guevara E11-254, 170517 Quito, Ecuador; 5 Infectious and Tropical Disease Institute, Department of Biomedical Sciences, Heritage College of Osteopathic Medicine, Ohio University, Athens, OH 45701 USA

**Keywords:** Vector-borne disease, Chagas disease, *Rhodnius ecuadoriensis*, *Sciurus stramineus*, *Simosciurus nebouxii*, Nest site preferences, Ecuador

## Abstract

**Background:**

The white-naped squirrel, *Simosciurus nebouxii* (previously known as *Sciurus stramineus*), has recently been identified as an important natural host for *Trypanosoma cruzi* in Ecuador. The nests of this species have been reported as having high infestation rates with the triatomine vector *Rhodnius ecuadoriensis*. The present study aims to determine the levels of nest infestation with *R. ecuadoriensis*, the ecological variables that are influencing the nest site selection, and the relationship between *R. ecuadoriensis* infestation and trypanosome infection.

**Results:**

The study was carried out in transects in forest patches near two rural communities in southern Ecuador. We recorded ecological information of the trees that harbored squirrel nests and the trees within a 10 m radius. Manual examinations of each nest determined infestation with triatomines. We recorded 498 trees (*n* = 52 with nests and *n* = 446 without nests). *Rhodnius ecuadoriensis* was present in 59.5% of the nests and 60% presented infestation with nymphs (colonization). Moreover, we detected *T. cruzi* in 46% of the triatomines analyzed.

**Conclusions:**

We observed that tree height influences nest site selection, which is consistent with previous observations of squirrel species. Factors such as the diameter at breast height and the interaction between tree height and tree species were not sufficient to explain squirrel nest presence or absence. However, the nest occupancy and tree richness around the nest were significant predictors of the abundance of triatomines. Nevertheless, the variables of colonization and infection were not significant, and the data observed could be expected because of chance alone (under the null hypothesis). This study ratifies the hypothesis that the ecological features of the forest patches around rural communities in southern Ecuador favor the presence of nesting areas for *S. nebouxii* and an increase of the chances of having triatomines that maintain *T. cruzi* populations circulating in areas near human dwellings. Additionally, these results highlight the importance of including ecological studies to understand the dynamics of *T. cruzi* transmission due to the existence of similar ecological and land use features along the distribution of the dry forest of southern Ecuador and northern Peru, which implies similar challenges for Chagas disease control.

**Electronic supplementary material:**

The online version of this article (10.1186/s13071-018-3138-4) contains supplementary material, which is available to authorized users.

## Background

Vector-borne diseases involve complex interactions among multiple sets of host, vector and pathogen species. Chagas disease is representative of such complex interactions. This disease is caused by the parasite *Trypanosoma cruzi* and is transmitted by the feces of triatomine bugs. In natural conditions, the parasite populations are maintained through systemic infections of mammalian hosts through a mechanic infection by skin contaminated with infected feces of triatomine bugs or by oral transmission due to the ingestion of infected vectors [1].

As in other host-parasite interactions, *T. cruzi* infections in vertebrate and invertebrate hosts occur in three overlapping and interchangeable cycles: domestic, peridomestic and sylvatic [2]. Although, these cycles have some particular characteristics, e.g. species more adapted to life inside the houses), the discrimination of these habitats is somewhat arbitrary, particularly with the peridomestic and sylvatic area boundaries. In general, the domestic cycle occurs when infected triatomines infest the human dwellings, e.g. bedrooms, kitchens, while the peridomestic cycle occurs when they infest man-built structures surrounding a house, e.g. chicken coops, piles of material. The sylvatic cycle occurs in vertebrate nests and burrows (used by birds and small mammals) in areas separate from what is defined as a human dwelling but where human activities can take place (e.g. crop fields, forest patches) [3, 4].

In southern Ecuador, the presence of parasites of the genus *Trypanosoma* (*T. cruzi* and *T. rangeli*) has been reported circulating in triatomine vectors and mammalian hosts [3–7]. The most abundant triatomine species in this region is *Rhodnius ecuadoriensis*, which is involved in all three transmission cycles (domestic, peridomestic and sylvatic) [4]. In the sylvatic cycle, an interesting association with the nests of the white-naped squirrel *Simosciurus nebouxii*, previously lumped with the Guayaquil squirrel *Sciurus stramineus* [8], has been reported [3, 9]. These squirrels, *S. nebouxii* and *S. stramineus*, have been identified as hosts for *T. cruzi* in coastal and southern Ecuador [3, 9–12]. They are considered important hosts because they inhabit areas surrounding human dwellings and their nests are suitable habitats for triatomines [13]. Additional evidence of a close relationship between *R. ecuadoriensis* and these squirrels was revealed by a spatial analysis that found that changes in land use and dispersal patterns of the squirrels influence triatomine abundance and *T. cruzi* persistence [12]. Therefore, the study of ecological dynamics of vector-host-pathogen interactions can provide novel insights into the mechanisms of emergence, maintenance and spread of diseases [14]. However, the interactions among invertebrate and vertebrate hosts and their influence on Chagas disease transmission have not yet been thoroughly evaluated. Notably, the understanding of ecological preferences, e.g. nesting habitat selection, of the squirrels is essential to assess vector and parasite dynamics, especially in areas where the increasing presence of human settlements near sylvatic environments challenges the effectiveness of Chagas disease control strategies.

The objectives of this study were to determine (i) the level of nest infestation of the squirrel *S. nebouxii* with the vector *R. ecuadoriensis*; (ii) the ecological variables that influence squirrel nest site selection; and (iii) the relationships between ecological variables and the abundance, colonization, and trypanosome infection of *R. ecuadoriensis* associated with squirrels.

## Results

Along the three transects of this study, a total of 498 trees were sampled. Of these, 52 trees presented one or more squirrel nests. Thirty species of trees [≥ 0.1 m of diameter at breast height (DBH)] were identified. *Vachellia macracantha* (*Fabaceae*, common name: faique) was the most abundant tree (38%), followed by 21% of *Pisonia aculeata* (*Nyctaginaceae*, common name: pego-pego) and 12% of *Morus celtidifolia* (*Moraceae*, common name: palo blanco). Fifteen percent of the sampled trees could not be identified (Additional file 1: Table S1).

### Entomological indices, population structure and natural trypanosome infection rates of triatomines

We examined 42 squirrel nests. Of these, 25 (60%) were infested with triatomines. We collected 298 individuals of *R. ecuadoriensis* from all infested nests. Overall triatomine density was 7.1 bugs per searched nest; crowding was 11.9 bugs per infested nest; and the overall colonization index was 60% (Table 1). Population structure analyses showed that nymphs III, IV and NV were more abundant than NI, NII and adults (females and males) (Table 1).

**Table 1:** Entomological indices and natural trypanosome infection rates of triatomines collected on squirrel’s nests in two rural communities in southern Ecuador

Community	Squirrel nests			Triatomines															
				Nymphs					Adults			Entomological indices				*Trypanosoma* infection (%)			
	*N*	Infested	With nymphs	I	II	III	IV	V	F	M	Total	In (%)	D	CW	C (%)	Total	TC	TR	Mx
Bellamaría	26	15	9	14	19	39	44	70	34	28	248	57.7	9.5	16.5	60	117	23.9	43.6	19.7
Bellamaría Chico	6	5	3	5	10	0	0	0	1	1	17	83.3	2.8	3.4	60	10	60.0	40.0	0.0
Chaquizcha	10	5	3	1	12	12	3	0	0	5	33	50.0	3.3	6.6	60	18	50.0	38.9	5.6
Total	42	25	15	20	41	51	47	70	35	34	298	59.5	7.1	11.9	60	145	29.7	42.8	16.6

*Abbreviations*: *F* female, *M* male, *In* infestation index, *D* density, *CW* crowding, *C* colonization, *TC* *T. cruzi*, *TR*, *T. rangeli*, *Mx* mixed infection *T. cruzi* + *T. rangeli*

We analyzed the infection with trypanosomes in a subset (145 individuals) of the collected triatomines. High infection rates were detected with *T. cruzi* (46%) and *T. rangeli* (59%), including mixed infection with both parasites (Table 1). Parasite infection was reported at all developmental stages.

### Predictors of nesting-habitat preferences of the white-naped squirrel

**Fig. 1:**
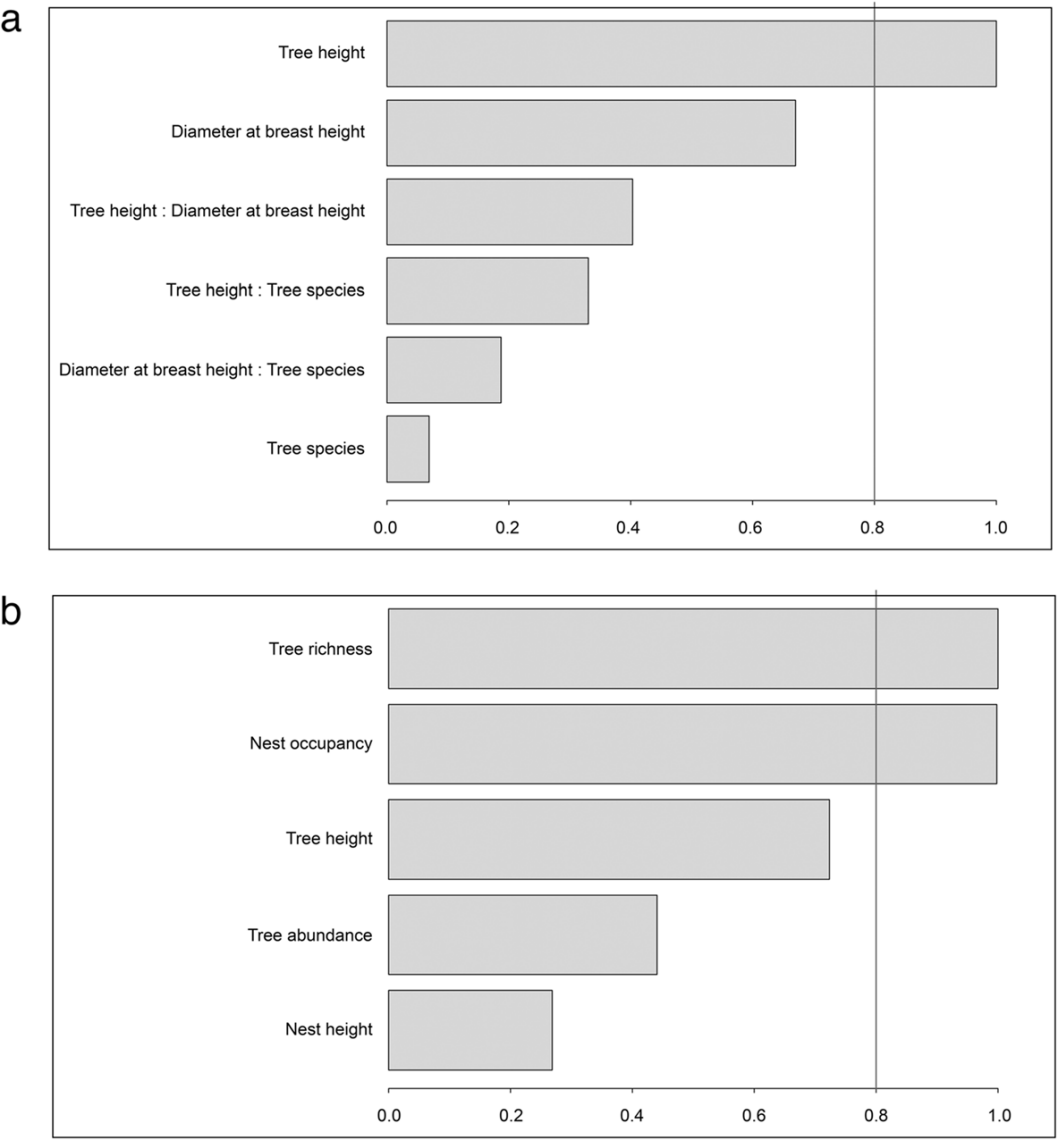
Multi-model inference analysis. Model-average importance of term results for nesting-habitat preferences of the squirrel (**a**) and effects of nests-habitat features on triatomine abundance (**b**)

We analyzed nesting-habitat preferences only for trees that had no missing values for any independent variable, totaling 310 trees, of which 44 harbored squirrel nests (Additional file 1: Table S1). Multi-model inference analysis revealed that the most parsimonious model for explaining squirrel nest presence/absence was one that included only tree height as an explanatory variable (AIC = 190.82) (Table 2). A model including tree height, diameter at breast height and the interaction between tree height and plant species also had good explanatory power, although with a slightly higher AIC (191.07) than just using tree height alone (Table 2). Finally, the model-averaged importance of term analysis revealed that tree height had the highest relative importance, followed by the diameter at breast height, and the interaction between the two variables, although only the former had importance higher than 80% (Fig. 1a).

**Table 2:** Results of the multi-model inference analysis of nest presence/absence, for the tree models with the lowest AIC

Model	AIC	∆ AIC^a^	LRT^b^	*P*-value
TH	190.82	73.68	66.30	0.60
TH + DBH + SPP:TH	191.07	73.43	16.54	1
TH + DBH + SPP:DBH	191.53	72.97	17.01	1

*Abbreviations*: *TH* tree height, *DBH* diameter at breast height, *SPP* tree species

^a^∆ AIC is the difference between the Akaike Information Criterion (AIC) for the complete model and the reduced model

^b^Likelihood ratio test (LRT) and associated *P*-value test the hypothesis that the reduced model provides no worse fit than the complete model

### Predictors of nests-habitat features on triatomine abundance, colonization and their infection with trypanosome species

To analyze the predictors of triatomine abundance, we included only trees with nests that had no missing data (*n* = 31) (Additional file 2: Table S2). Model inference analyses revealed that the most parsimonious model was one that included only nest occupancy, tree height, and tree richness around the nest (Table 3). Also, according to the model-averaged importance of term analysis, nest occupancy and tree richness around the nest were both equally important and had very high support. Tree height also had high support, although lower than the other two variables (Fig. 1b).

**Table 3:** Results of the multi-model inference analysis on triatomine abundance, for the tree models with the lowest AIC_c_

Model	AIC_c_	∆ AIC_c_^a^	LRT^b^	*P*-value
NO + TH + TR	609.33	2.81	3.15	0.21
NO + TH + TA + TR	610.08	2.06	1.05	0.31
NO + NH + TH + TR	611.21	0.93	2.18	0.14

*Abbreviations*: *NO* nest occupancy, *TH* tree height, *TR* tree richness, *TA* tree abundance, *NH* nest height

^a^∆ AIC_c_ is the difference between the corrected Akaike Information Criterion (AIC_c_) for the complete model and the reduced model

^b^Likelihood ratio test (LRT), and associated *P*-value test the hypothesis that the reduced model provides no worse fit than the complete model

The analysis of the predictors of triatomine colonization and their infection with trypanosome species was calculated for trees with the presence of both nest and triatomines (*n* = 20) (Additional file 2: Table S2). The analyses found that no model explains the response better than a null model and that none of the explanatory variables has a significant effect on the response.

## Discussion

The high triatomine infestation rates and densities of vectors found in this study confirm the white-naped squirrel (*S. nebouxii*) as an important natural food source for triatomines in the southern Andean region of Ecuador [3, 9]. Despite the extensive distribution of the vector *R. ecuadoriensis*, its presence in different habitats and its close association with other arboreal vertebrates, e.g. birds, rodents) [3], reaches its highest abundance in squirrel (*S. nebouxii*) nests [3, 9, 10, 12]. Also, other squirrel species of the genus *Sciurus* and the vector extensively overlap in both range and habitat, across the equatorial Pacific region of western Ecuador and northwestern Peru between 0–2000 masl [8, 15].

Our results reveal that ecological features of squirrel nests, such as tree height and tree richness in the surrounding forest, could favor the presence of triatomines. Although *R. ecuadoriensis* may not be specialized to *S. nebouxii*, ecological and environmental conditions might favor an overlap in geographical distribution of the two species [13, 16, 17], and thus, their interaction. Nevertheless, the high infestation rates could suggest that *S. nebouxii* favors *R. ecuadoriensis* population persistence in relation to other sympatric vertebrate hosts, where conditions are suitable. Furthermore, phenotypic variability in *R. ecuadoriensis* [17] could be a critical factor in the optimal exploitation of all available resources (i.e. host species) for successful vector colonization and multiplication [18].

### Infestation of squirrel nest with triatomines

We detected a high infection rate (60%) and found only one species (*R. ecuadoriensis*) of the four species of triatomine reported for southern Ecuador. This finding is in accordance with other reports of *R. ecuadoriensis* (instars and adults) in sylvatic environments associated with squirrel nests. However, the infestation rate is higher than previously reported: 14% [9] and 12% [3].

In this study, triatomine population structure, with the presence of all nymphal stages and adults, revealed long-term colonization of triatomines. Considering that temperature influences the duration of triatomine life-cycle [19] and that *R. ecuadoriensis* requires around six months to reach the adult stage [20], the presence of nymphs could thus indicate stable conditions for triatomines in squirrel nests. The effectiveness of triatomines at colonizing nests of a particular host species could be primarily related to adult oviposition rate, offspring performance [21] and availability of at least one blood meal to molt [20]. Survival under those conditions and within a seasonal environment, with a mortality risk during nymphal development, could be significantly maximized by biotic (e.g. protection against predation, intra- and interspecific competition) and abiotic (e.g. adequate temperature and humidity conditions) features of squirrel nest.

### Trypanosome infection of triatomines

We detected *T. cruzi* and *T. rangeli* in 89% of the analyzed triatomines, of which more than half were nymphs (57%). The higher trypanosome prevalence in nymphs, along with nymph limited dispersal capacity [22, 23], suggests that they acquired the parasites from infected squirrels and/or the opportunistic rodents that use the abandoned nests. The effective parasite transmission could be related to the primary infection route through contact with triatomine feces (i.e. stercorarian transmission). However, the most effective pathway of infection for wildlife in sylvatic cycles is predation on infected bugs, as it happens in other natural hosts (e.g. raccoons, opossums) [24]. Nevertheless, mammalian host immunity should be further explored in order to understand the influence of trypanosome parasitemia and the degree of tolerance to repeated triatomine exposure.

### Selection of nesting sites by squirrels

Some species of tree squirrels, including the white-naped squirrel, build nests on trees. Nests are important resources, and they are used for sleeping, resting, and provide protection against weather conditions and predators, and serve as places to raise offspring [25, 26]. *Simosciurus nebouxii* nests are loosely constructed of woven sticks of about 30 cm in diameter [13]. Nesting site selection depends on many factors (i.e. position, biome composition, the height of the tree) and can be a limiting factor for squirrel distribution [27]. Our results demonstrated that tree height (TH) is an important variable for nesting site selection of *S. nebouxii*, together with the diameter at breast height (DBH), although to a lesser extent. These findings are in accordance with other studies of tree squirrels (i.e. Albert’s squirrel, Virginia northern flying squirrel) that reported TH and DBH as important variables for nesting site selection [27, 28].

In this analysis, tree species did not explain the absence or presence of squirrel nests. Only 30 species of trees (≥ 0.1 m of DBH) were identified. Of these, 22 species are higher than 800 cm up to almost 30 meters. Despite the diversity of tree species, only two species (*Vachellia macracantha* and *Pisonia aculeata*) accounted for 57% of trees and harbored the 48% of squirrels’ nests. The selection of a nesting site could explain this result is more related to the availability of trees than to a specific preference for tree species. In other squirrel species (e.g. Albert’s squirrel), tree size and access routes appear to be more important to the selection of nest sites than tree species [28].

### Features of nesting habitat in relation to triatomine abundance and trypanosome presence

Wild and synanthropic rodents are important for *T. cruzi* transmission in several regions; however, their role varies with time and place [29]. Biotic factors (i.e. nutritional status, age, stress conditions, abundance) are important in host-vector-parasite interaction [30] and the particular association of squirrel-triatomines-*T. cruzi* has been previously reported [3, 10, 12, 31, 32]. Nevertheless, little information is available about the ecological characteristics of this association.

Multi-model inference analysis revealed that tree height, nest occupancy and tree richness influence the presence of triatomines in squirrel nests. The importance of tree height has been demonstrated in previous studies that reported higher triatomine abundance in squirrel nests located five meters above ground level and close to human dwellings [3, 10]. At the same time, nest occupancy is an important factor due to the availability of blood, which is essential for triatomine development [20, 33]. In sylvatic environments as well as in environments near human dwellings, squirrels are not the only available host. Other arboreal rodents could, eventually, use the abandoned squirrel nest and serve as blood sources for triatomines. Within this context, the importance of tree richness might have an impact on vertebrate host diversity and the dynamics on rodents, which implies opportunistic behavior of other rodent species to use the squirrel nests and serve as an alternative blood source for triatomines.

Despite the role of other rodent species, it has been demonstrated that squirrels have a very important impact on triatomine abundance and distribution. Previous studies in sylvatic environment reported a triatomine infestation rate > 14% in squirrel nests, and much lower infestation rates in other habitats, such as bird and mouse/rat nests [3, 9]. Additionally, it has been shown that triatomines are associated with squirrels all year round and closely related to human activities such as cultivation of maize. Therefore, land use constitutes an important factor in the dispersal patterns of sylvatic triatomines, particularly of *R. ecuadoriensis*, which is temporally and spatially more closely related to squirrel dynamics than those of other available hosts [12]. Moreover, land use impact might also be reflected in the presence of a pathogen within the host species, depending on the specific biology of the host-parasite relationship [34, 35]. Also, as important as the association of this squirrel species with triatomine abundance, it would be important to unveil the side effects of the use of abandoned nest by opportunistic rodents because of the generation of new habitats.

## Conclusions

The interaction between hosts (vertebrate and invertebrate) and parasites represents a complex scenario. This study revealed that particular ecological characteristics of the white-naped squirrel (*S. nebouxii)* have local implications for the maintenance of triatomines and trypanosomes and thus, constitute a risk factor to consider when there are near human settlements. Indeed, these results might be considered when assessing transmission risk of *T. cruzi* in areas with similar ecological and land use features along the distribution of the dry forest in southern Ecuador and northern Peru, which might face similar challenges when triatomine control strategies are applied. This study corroborates *S. nebouxii* as the main factor for the maintenance of *T. cruzi* circulating in areas near human dwellings. Moreover, squirrel presence favors the preservation of triatomine populations, especially *R. ecuadoriensis*, that may, eventually, colonize human dwellings. Further studies on the ecology of vertebrate hosts are essential for understanding the dynamics of *T. cruzi* transmission, especially in areas where classic control strategies have shown limited effectiveness. Therefore, long-term Chagas disease control strategies must consider the ecological characteristics of the surrounding areas because those represent a source of triatomines that can invade human dwellings. In this regard, house improvement activities in order to generate physical barriers for the entrance of triatomines, the detection of possible sources of triatomines in forest patches around the houses, together with the involvement of the community in prevention activities and surveillance are key for a sustainable strategy to control Chagas disease transmission.

## Methods

### Study area

**Fig. 2:**
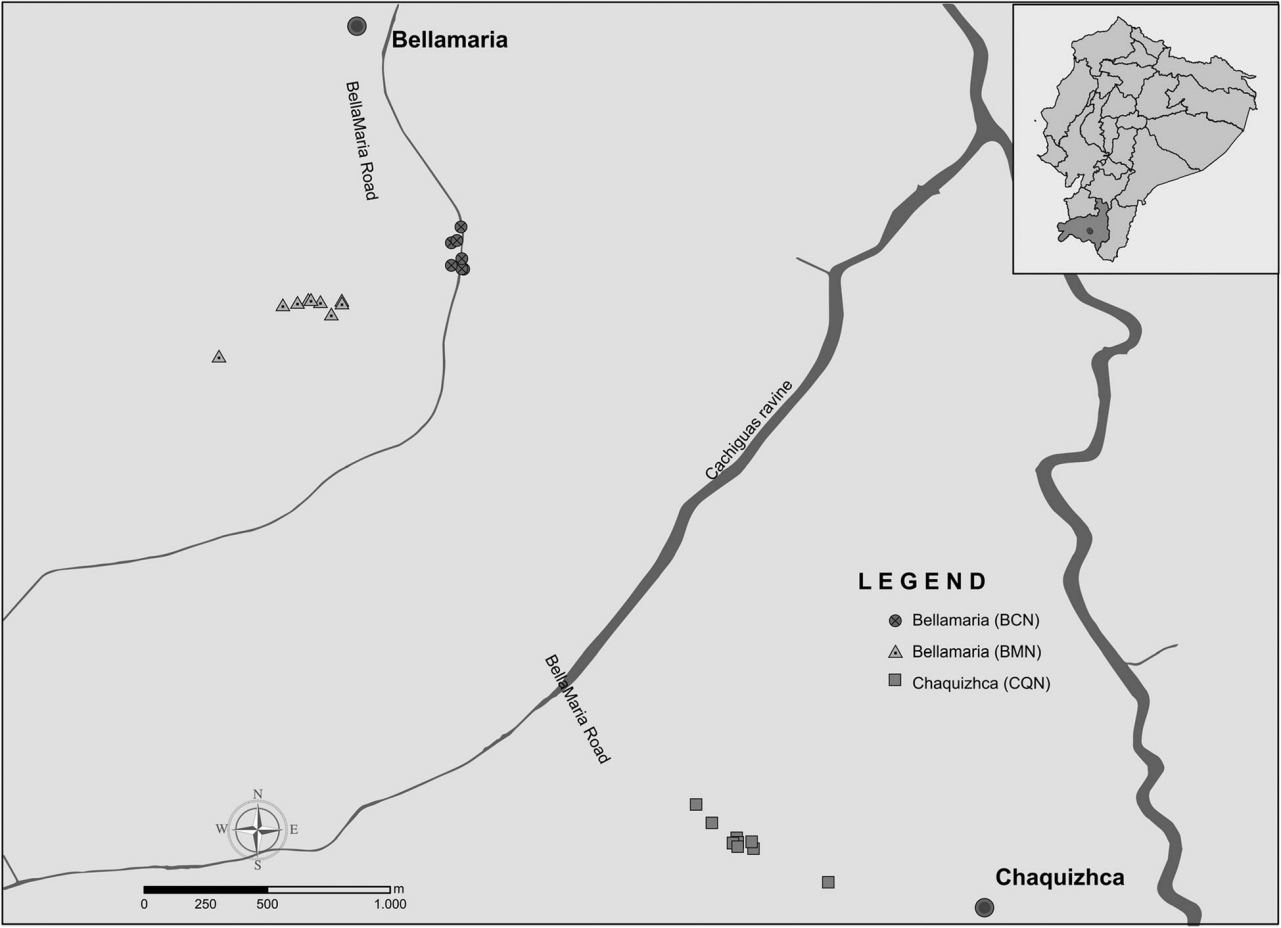
Location of sampled squirrel nests along the transects in the communities of Bellamaria and Chaquizhca in southern Ecuador

We carried out data collection during June and July of 2012 and 2013. The study area includes three forest fragments, in two rural communities of Loja Province, located on the slopes of the southwestern Ecuadorian Andes: Bellamaria (4°11'27.6"S, 79°37'15.599"W; 1150 meters above sea level, masl) and Chaquizhca (4°13'30"S, 79°35'52.799"W; 1162 masl) (Fig. 2). These communities were selected based on previous reports of high infection rates with trypanosomatids of sylvatic *Rhodnius ecuadoriensis* populations, associated with *Simosciurus nebouxii* nests [3, 9], and also due to ease of access and permission by owners.

Within the study area, households are scattered amongst a mosaic of small forest fragments surrounded by crop plantations (maize, kidney beans, yucca, coffee and peanuts), pastures, and dirt roads [3]. The study area is part of the neotropical, seasonal, dry forest and has vegetation of both the Central Andes Coast and Central Inter-Andean Valleys floristic groups [36]. Its native vegetation is dominated by *Cedrela fissilis* (*Meliaceae*; Spanish name: cedro), *Vachellia macracantha* (*Fabaceae*; Spanish name: faique) and *Pisonia aculeata* (*Nyctaginaceae*; Spanish name: pego-pego).

### Data collection

We followed a transect approximately one-kilometer-long in each forest fragment included in the study (two transects in Bellamaria and one in Chaquizhca) (Fig. 2). Along each transect, all the trees that harbored squirrels’ nests were georeferenced and assigned a field code. For all those trees, we collected the following variables: tree species (SPP) (samples were collected for taxonomic identification at the QCA herbarium at PUCE), tree height (TH, which was measured with a clinometer model PM-5/1520, Suunto, Vantaa, Finland), tree diameter at breast height (DBH, measured at 1.30 m above the ground with a measuring tape), height of the nest on the tree (NH) and nest occupancy (NO), defined as the presence of a squirrel in the nest at the time of inspection or conclusive evidence of current occupancy (e.g. urine odor, presence of fur). Additionally, information about SPP, TH, DBH, tree species richness (TR) and abundance (TA) was recorded within a 10 m radius around the focal tree (note that we only included trees ≥ 0.1 m of DBH).

### Triatomine searches

We conducted triatomine searches on the nests that could be taken from tree branches. A search effort of 10 min/person/nest was performed following all safety standards as previously described [10]. A nest was considered positive when at least one live triatomine of any developmental stage was found. The triatomines were placed in labeled plastic containers and transported to the insectary at the Center for Research on Health for Latin America at Pontifical Catholic University of Ecuador (CISeAL, PUCE) for species identification and stage classification (NI-NV for nymphal stages or male/female for adults).

### Entomological indices

We estimated entomological indices with four standard calculations, according to the WHO recommendations [37] (i) infestation rate (IIn): number of infested nests/number of searched nests × 100; (ii) density (D): number of captured triatomines/number of searched nests; (iii) crowding (CW): number of captured triatomines/number of infested nests; and colonization index (C): nests with presence of nymphs/number of infested nests × 100.

### Natural infection of triatomines with trypanosomes

Intestinal contents from captured triatomines were isolated for detection of trypanosomes. Detection was carried through PCR amplification of the conserved domain of minicircle of kinetoplast DNA (kDNA), as described in [6, 38]. The differential detection of *T. cruzi* and *T. rangeli* was based on the size of the PCR products. A band of 330 bp was expected for *T. cruzi*, whereas a band of 760 bp together with bands of 300–450 bp defined *T. rangeli*. Infection rates for *T. cruzi* and *T. rangeli* were calculated by dividing the number of positive samples by the total number of analyzed samples.

### Nesting habitat preferences of the white-naped squirrel *S. nebouxii*

We used a multi-model inference approach to test the effect of TH, DBH and SPP on the presence or absence of squirrel nests (NEST). We fitted binomial, generalized linear models (GLM, containing all possible subsets of the explanatory variables and all the possible interactions between them) with a logit link function, using the *glmulti* package of R [39]. The most parsimonious model was identified using the Akaike Information Criterion (AIC). Also, we used likelihood ratio tests (with the *lrtest* function of the *lmtest* package of R) to analyze the difference between the null model (with only an intercept, NEST ~ 1) and the fitted models in order to determine if the fitted models predicted the response significantly better than by chance. Finally, the *glmulti* package also allowed us to determine the relative importance of the various independent variables, by summing the weights/probabilities of the models in which each variable appeared. These values can be regarded as the overall support for each variable across all models [40]. The dataset used for this analysis is detailed in Additional file 1: Table S1.

### Effects of nest-habitat features on triatomine abundance, colonization and their infection with trypanosome species

We followed a similar approach (multi-model inference) to understand which variables explain triatomine abundance (ABUND), colonization (COL, presence/absence of nymphs) and infection by trypanosome species (INF). We fitted GLMs to test the effect of tree height (TH), nest height (NH), nest occupancy (NO), tree richness (TR) and tree abundance (TA) on each response variable. In the case of triatomine abundance, we fitted Poisson log-linear models. For the other two response variables, we fitted binomial models with a logit link function. We did not include interactions for these analyses because the number of possible models became too large and the resulting models were too complex and difficult to interpret. The more parsimonious model was identified using the corrected Akaike Information Criterion (AIC_c_), which is recommended for small sample sizes (see [41]). Also, we used likelihood ratio tests to analyze the difference between null models and the fitted models. Finally, as for nest presence/absence, we determined the relative importance of the various model variables (see above). The dataset used for this analysis is detailed in Additional file 2: Table S2.

## Additional files

Additional file 1: **Table S1.** Tree diversity and presence of squirrel nests. Multi-model inference data used to evaluate the preference of nesting site based on the ecological characteristics of the presence or absence of squirrel nests. (DOCX 46 kb)

Additional file 2: **Table S2.** Effect on nest-habitat features. Multi-model inference data used to evaluate the effects on triatomine abundance, colonization and their infection with *T. cruzi*. (DOCX 18 kb)

## Abbreviations

ABUND: Triatomine abundance
AIC: Akaike Information Criterion
C: Colonization index
COL: Presence/absence of nymphs
CW: Crowding
D: Density
DBH: Tree diameter at breast height
GLM: Generalized Linear Model
IIn: Infestation rate
INF: Infection with trypanosomes
kDNA: Kinetoplast DNA
masl: Meters above sea level
NEST: Presence or absence of squirrel nest
NH: Height of nest on the tree
NO: Nest occupancy
SPP: Tree species
TA: Tree abundance
TH: Tree height
TR: Tree species richness
