# Supplementary material for: Influence of ecological factors on the presence of a triatomine species associated with the arboreal habitat of a host of Trypanosoma cruzi
Source: Parasit Vectors. 2018 Oct 29;11:567. doi: 10.1186/s13071-018-3138-4 (PMC6206927; doi:10.1186/s13071-018-3138-4)
Supplement: Supplementary file 1 — Table S1. Tree diversity and presence of squirrel nests. Multi-model inference data used to evaluate the preference of nesting site based on the ecological characteristics of the presence or absence of squirrel nests. (DOCX 46 kb) [file 13071_2018_3138_MOESM1_ESM.docx]

**Additional file 1**

**Table S1.** Tree diversity and presence of squirrel nests. Multi-model inference data used to evaluate the preference of nesting site based on the ecological characteristics of the presence or absence of squirrel nests.

| **Tree code** | **NEST^a^** | **SPP** | **Family** | **DBH (cm)** | **TH**  **(cm)** |
| --- | --- | --- | --- | --- | --- |
| BCN01^b^ | 1 | *Pisonia aculeata* | Nyctaginaceae | 57.40 | 964 |
| BCN02^b^ | 1 | *Vachellia macracantha* | Fabaceae | 72.40 | 1,089 |
| BCN03^b^ | 1 | *Vachellia macracantha* | Fabaceae | 45.00 | 1255 |
| BCN04^b^ | 1 | *Vachellia macracantha* | Fabaceae | 23.00 | 2,000 |
| BCN05^b^ | 1 | *Morus celtidifolia* | Moraceae | 90.30 | 1,462 |
| BCN06^b^ | 1 | *Agonandra excelsa* | Opiliaceae | 22.6 | 651 |
| BMN01^b^ | 1 | *Aegiphyla* cf. | Lamiaceae | 87 | 1,200 |
| BMN02^b^ | 1 | *Vachellia macracantha* | Fabaceae | 146 | 2,653 |
| BMN03^b^ | 1 | *Vachellia macracantha* | Fabaceae | 23 | 997 |
| BMN04^b^ | 1 | *Morus celtidifolia* | Moraceae | 230 | 1,600 |
| BMN05^b^ | 1 | *Vachellia macracantha* | Fabaceae | 100 | 850 |
| BMN06^b^ | 1 | *Cedrela fissilis* | Meliaceae | 85 | 900 |
| BMN07^b^ | 1 | *Morus celtidifolia* | Moraceae | 67 | 1,400 |
| BMN08^b^ | 1 | *Vachellia macracantha* | Fabaceae | 99 | 800 |
| BMN09^b^ | 1 | *Pisonia aculeata* | Nyctaginaceae | 177 | 1,389 |
| BMN10^b^ | 1 | *Vachellia macracantha* | Fabaceae | 105 | 1,161 |
| BMN11^b^ | 1 | *Ziziphus thyrsiflora* | Rhamnaceae | 230.5 | 1,400 |
| BMN12^b^ | 1 | *Ziziphus thyrsiflora* | Rhamnaceae | 230.5 | 1,400 |
| BMN13^b^ | 1 | *Ziziphus thyrsiflora* | Rhamnaceae | 230.5 | 1,400 |
| BMN14 | 1 | NA | NA | NA | 1,439 |
| BMN15 | 1 | NA | NA | NA | 1,439 |
| BMN16^b^ | 1 | *Cedrela fissilis* | Meliaceae | 94.5 | 1,264 |
| BMN17 | 1 | NA | NA | NA | 2,964 |
| BMN18 | 1 | NA | NA | NA | 1,764 |
| BMN19 | 1 | NA | NA | NA | 2,964 |
| BMN20 | 1 | NA | NA | NA | 2,964 |
| BMN21^b^ | 1 | *Geoffroea spinosa* | Fabaceae | 75.9 | 2,864 |
| BMN22 | 1 | NA | NA | NA | 1,764 |
| BMN23 | 1 | NA | NA | NA | 1,564 |
| BMN24^b^ | 1 | *Pisonia aculeata* | Nyctaginaceae | 92 | 1,114 |
| BMN25^b^ | 1 | *Geoffroea spinosa* | Fabaceae | 84.7 | 864 |
| BMN26^b^ | 1 | *Vachellia macracantha* | Fabaceae | 86.8 | 1,164 |
| BMN27^b^ | 1 | *Pisonia aculeata* | Nyctaginaceae | 233.4 | 1,274 |
| BMN28 | 1 | *Pisonia aculeata* | Nyctaginaceae | 75.6 | NA |
| BMN29 | 1 | *Eriotheca ruizii* | Malvaceae | 70.08 | NA |
| BMN30 | 1 | *Senna mollissima* | Fabaceae | 154 | NA |
| BMN31^b^ | 1 | *Eriotheca ruizii* | Malvaceae | 22.8 | 1,064 |
| BMN32^b^ | 1 | *Morus celtidifolia* | Moraceae | 118.9 | 1,614 |
| BMN33^b^ | 1 | *Vachellia macracantha* | Fabaceae | 70 | 1,064 |
| BMNR01^b^ | 1 | *Aegiphyla cf* | Lamiaceae | 87 | 1,200 |
| BMNR09^b^ | 1 | *Pisonia aculeata* | Nyctaginaceae | 177 | 1,389 |
| BMNR10^b^ | 1 | *Vachellia macracantha* | Fabaceae | 105 | 1,161 |
| CQN01^b^ | 1 | *Pisonia aculeata* | Nyctaginaceae | 187 | 655 |
| CQN02^b^ | 1 | *Vachellia macracantha* | Fabaceae | 188 | 1,370 |
| CQN03^b^ | 1 | *Vachellia macracantha* | Fabaceae | 105 | 1,510 |
| CQN04^b^ | 1 | *Pisonia aculeata* | Nyctaginaceae | 36.1 | 960 |
| CQN05^b^ | 1 | *Vachellia macracantha* | Fabaceae | 135 | 830 |
| CQN06^b^ | 1 | *Morus celtidifolia* | Moraceae | 93 | 1,100 |
| CQN07^b^ | 1 | *Pisonia aculeata* | Nyctaginaceae | 140 | 1,048 |
| CQN08^b^ | 1 | *Morus celtidifolia* | Moraceae | 69.4 | 900 |
| CQN09^b^ | 1 | *Pisonia aculeata* | Nyctaginaceae | 141 | 1,180 |
| CQN10^b^ | 1 | *Pisonia aculeata* | Nyctaginaceae | 129 | 1,100 |
| BCN01_1 | 0 | NA | NA | 99 | 700 |
| BCN01_2^b^ | 0 | *Morus celtidifolia* | Moraceae | 33.8 | 420 |
| BCN01_3 ^b^ | 0 | Undetermined sp.1 | NA | 64.7 | 810 |
| BCN01_4^b^ | 0 | *Morus celtidifolia* | Moraceae | 126 | 1,400 |
| BCN01_5^b^ | 0 | *Vachellia macracantha* | Fabaceae | 50 | 700 |
| BCN01_6^b^ | 0 | *Vachellia macracantha* | Fabaceae | 295 | 1,000 |
| BCN02_1^b^ | 0 | *Vachellia macracantha* | Fabaceae | 27.6 | 800 |
| BCN02_2^b^ | 0 | *Vachellia macracantha* | Fabaceae | 19.3 | 730 |
| BCN02_3^b^ | 0 | *Vachellia macracantha* | Fabaceae | 26.8 | 750 |
| BCN02_5^b^ | 0 | *Vachellia macracantha* | Fabaceae | 36.5 | 600 |
| BCN02_6^b^ | 0 | *Vachellia macracantha* | Fabaceae | 41.4 | 600 |
| BCN02_7^b^ | 0 | *Vachellia macracantha* | Fabaceae | 21.8 | 700 |
| BCN02_8^b^ | 0 | *Vachellia macracantha* | Fabaceae | 30.5 | 700 |
| BCN02_9^b^ | 0 | *Vachellia macracantha* | Fabaceae | 26.7 | 700 |
| BCN02_10^b^ | 0 | *Vachellia macracantha* | Fabaceae | 19 | 820 |
| BCN02_11^b^ | 0 | *Vachellia macracantha* | Fabaceae | 32 | 450 |
| BCN02_12^b^ | 0 | *Ceiba insignis* | Malvaceae | 586 | 1,620 |
| BCN02_13^b^ | 0 | *Morus celtidifolia* | Moraceae | 48.4 | 600 |
| BCN02_14^b^ | 0 | *Ipomoea* sp.1 | Convolvulaceae | 93 | 850 |
| BCN02_15^b^ | 0 | *Vachellia macracantha* | Fabaceae | 52 | 730 |
| BCN02_16^b^ | 0 | *Vachellia macracantha* | Fabaceae | 41.6 | 510 |
| BCN02_17^b^ | 0 | *Vachellia macracantha* | Fabaceae | 41.5 | 700 |
| BCN02_18^b^ | 0 | *Vachellia macracantha* | Fabaceae | 37.8 | 620 |
| BCN02_19^b^ | 0 | *Vachellia macracantha* | Fabaceae | 22.2 | 600 |
| BCN02_20^b^ | 0 | *Vachellia macracantha* | Fabaceae | 39.6 | 1,000 |
| BCN02_21^b^ | 0 | *Vachellia macracantha* | Fabaceae | 28.7 | 750 |
| BCN02_22^b^ | 0 | *Vachellia macracantha* | Fabaceae | 19.4 | 700 |
| BCN03_1^b^ | 0 | *Vachellia macracantha* | Fabaceae | 26.7 | 700 |
| BCN03_2^b^ | 0 | *Vachellia macracantha* | Fabaceae | 19 | 820 |
| BCN03_3^b^ | 0 | *Vachellia macracantha* | Fabaceae | 32 | 450 |
| BCN03_4^b^ | 0 | *Vachellia macracantha* | Fabaceae | 26.8 | 750 |
| BCN03_5^b^ | 0 | *Vachellia macracantha* | Fabaceae | 16.5 | 450 |
| BCN03_6^b^ | 0 | *Vachellia macracantha* | Fabaceae | 19.8 | 600 |
| BCN03_7^b^ | 0 | *Vachellia macracantha* | Fabaceae | 24 | 800 |
| BCN03_8^b^ | 0 | *Vachellia macracantha* | Fabaceae | 30.5 | 700 |
| BCN03_9^b^ | 0 | *Vachellia macracantha* | Fabaceae | 21.8 | 700 |
| BCN03_10^b^ | 0 | *Vachellia macracantha* | Fabaceae | 41.4 | 600 |
| BCN03_11^b^ | 0 | *Vachellia macracantha* | Fabaceae | 27.6 | 800 |
| BCN03_12^b^ | 0 | *Vachellia macracantha* | Fabaceae | 36.5 | 600 |
| BCN03_13^b^ | 0 | *Vachellia macracantha* | Fabaceae | 18.4 | 680 |
| BCN03_14 | 0 | NA | NA | 22 | 500 |
| BCN03_15^b^ | 0 | *Vachellia macracantha* | Fabaceae | 39.6 | 1,000 |
| BCN03_16^b^ | 0 | *Vachellia macracantha* | Fabaceae | 19.3 | 730 |
| BCN03_17 | 0 | NA | NA | 37.4 | 700 |
| BCN03_18^b^ | 0 | *Vachellia macracantha* | Fabaceae | 40 | 750 |
| BCN03_19^b^ | 0 | *Vachellia macracantha* | Fabaceae | 32.8 | 520 |
| BCN03_20^b^ | 0 | *Vachellia macracantha* | Fabaceae | 28.8 | 470 |
| BCN03_21^b^ | 0 | *Vachellia macracantha* | Fabaceae | 14 | 500 |
| BCN03_22 | 0 | NA | NA | 22 | 620 |
| BCN03_23^b^ | 0 | *Vachellia macracantha* | Fabaceae | 22.2 | 600 |
| BCN03_24^b^ | 0 | *Vachellia macracantha* | Fabaceae | 19.4 | 700 |
| BCN03_25^b^ | 0 | *Vachellia macracantha* | Fabaceae | 28.7 | 750 |
| BCN03_26^b^ | 0 | *Vachellia macracantha* | Fabaceae | 37.8 | 620 |
| BCN03_27^b^ | 0 | *Vachellia macracantha* | Fabaceae | 41.5 | 700 |
| BCN03_28^b^ | 0 | *Vachellia macracantha* | Fabaceae | 41.6 | 510 |
| BCN03_29^b^ | 0 | *Vachellia macracantha* | Fabaceae | 72.4 | 1,089 |
| BCN03_30^b^ | 0 | *Vachellia macracantha* | Fabaceae | 52 | 730 |
| BCN04_1^b^ | 0 | *Vachellia macracantha* | Fabaceae | 16.8 | 650 |
| BCN04_2^b^ | 0 | *Vachellia macracantha* | Fabaceae | 36 | 750 |
| BCN04_3^b^ | 0 | *Vachellia macracantha* | Fabaceae | 22.4 | 720 |
| BCN04_4^b^ | 0 | *Vachellia macracantha* | Fabaceae | 12 | 400 |
| BCN04_5^b^ | 0 | *Vachellia macracantha* | Fabaceae | 24.2 | 700 |
| BCN04_6^b^ | 0 | *Vachellia macracantha* | Fabaceae | 16 | 530 |
| BCN04_7^b^ | 0 | *Vachellia macracantha* | Fabaceae | 16 | 620 |
| BCN04_8^b^ | 0 | *Vachellia macracantha* | Fabaceae | 29.6 | 850 |
| BCN04_9^b^ | 0 | *Vachellia macracantha* | Fabaceae | 17 | 500 |
| BCN04_10^b^ | 0 | *Vachellia macracantha* | Fabaceae | 32.6 | 850 |
| BCN04_11^b^ | 0 | *Vachellia macracantha* | Fabaceae | 19 | 900 |
| BCN04_12^b^ | 0 | *Vachellia macracantha* | Fabaceae | 10.6 | 680 |
| BCN04_13^b^ | 0 | *Vachellia macracantha* | Fabaceae | 24.2 | 800 |
| BCN04_14^b^ | 0 | *Vachellia macracantha* | Fabaceae | 26.7 | 800 |
| BCN04_15^b^ | 0 | *Vachellia macracantha* | Fabaceae | 27.6 | 700 |
| BCN04_16^b^ | 0 | *Vachellia macracantha* | Fabaceae | 32.4 | 1,000 |
| BCN04_17^b^ | 0 | *Ceiba insignis* | Malvaceae | 19.02 | 1,500 |
| BCN04_18^b^ | 0 | *Erythrina* sp. | Fabaceae | 112 | 1,300 |
| BCN04_19^b^ | 0 | *Geoffroea spinosa* | Fabaceae | 16.7 | 430 |
| BCN04_20^b^ | 0 | *Ipomoea pauciflora* | Convolvulaceae | 38.8 | 700 |
| BCN04_21^b^ | 0 | *Vachellia macracantha* | Fabaceae | 44.6 | 700 |
| BCN04_22^b^ | 0 | *Vachellia macracantha* | Fabaceae | 27.2 | 522 |
| BCN04_23^b^ | 0 | *Vachellia macracantha* | Fabaceae | 17.6 | 500 |
| BCN04_24^b^ | 0 | *Ficus* sp. | Moraceae | 180 | 1,100 |
| BCN04_26^b^ | 0 | *Vachellia macracantha* | Fabaceae | 17 | 1,060 |
| BCN04_27^b^ | 0 | *Vachellia macracantha* | Fabaceae | 11.27 | 800 |
| BCN04_28^b^ | 0 | *Vachellia macracantha* | Fabaceae | 20 | 900 |
| BCN04_29^b^ | 0 | *Vachellia macracantha* | Fabaceae | 16.6 | 820 |
| BCN04_30 | 0 | NA | NA | 23.2 | 850 |
| BCN04_31^b^ | 0 | *Vachellia macracantha* | Fabaceae | 55 | 800 |
| BCN04_32^b^ | 0 | *Vachellia macracantha* | Fabaceae | 72 | 900 |
| BCN04_33^b^ | 0 | *Vachellia macracantha* | Fabaceae | 23.2 | 700 |
| BCN04_34^b^ | 0 | *Vachellia macracantha* | Fabaceae | 21.8 | 630 |
| BCN04_35^b^ | 0 | *Vachellia macracantha* | Fabaceae | 22.4 | 700 |
| BCN04_36^b^ | 0 | *Vachellia macracantha* | Fabaceae | 17.8 | 650 |
| BCN04_37 | 0 | NA | NA | 15.2 | 600 |
| BCN04_38^b^ | 0 | *Vachellia macracantha* | Fabaceae | 21 | 650 |
| BCN04_39^b^ | 0 | *Vachellia macracantha* | Fabaceae | 18.8 | 700 |
| BCN04_40^b^ | 0 | *Vachellia macracantha* | Fabaceae | 16.5 | 630 |
| BCN04_41^b^ | 0 | *Vachellia macracantha* | Fabaceae | 16 | 600 |
| BCN04_42^b^ | 0 | *Vachellia macracantha* | Fabaceae | 19 | 600 |
| BCN05_1^b^ | 0 | *Vachellia macracantha* | Fabaceae | 37 | 800 |
| BCN05_3 | 0 | *Morus celtidifolia* | Moraceae | NA | 1200 |
| BCN05_4 | 0 | NA | NA | 50.2 | 600 |
| BCN05_5^b^ | 0 | *Vachellia macracantha* | Fabaceae | 27.6 | 700 |
| BCN05_6^b^ | 0 | *Geoffroea spinosa* | Fabaceae | 16.7 | 430 |
| BCN05_7^b^ | 0 | *Ipomoea pauciflora* | Convolvulaceae | 38.8 | 700 |
| BCN05_8^b^ | 0 | *Vachellia macracantha* | Fabaceae | 44.6 | 700 |
| BCN05_9^b^ | 0 | *Vachellia macracantha* | Fabaceae | 27.2 | 522 |
| BCN05_10^b^ | 0 | *Vachellia macracantha* | Fabaceae | 23 | 2000 |
| BCN05_11^b^ | 0 | *Vachellia macracantha* | Fabaceae | 16.6 | 820 |
| BCN05_12^b^ | 0 | *Vachellia macracantha* | Fabaceae | 20 | 900 |
| BCN05_13^b^ | 0 | *Vachellia macracantha* | Fabaceae | 11.27 | 800 |
| BCN05_14^b^ | 0 | *Vachellia macracantha* | Fabaceae | 17 | 1060 |
| BCN05_15^b^ | 0 | *Vachellia macracantha* | Fabaceae | 55 | 800 |
| BCN05_16^b^ | 0 | *Vachellia macracantha* | Fabaceae | 72 | 900 |
| BCN05_17^b^ | 0 | *Vachellia macracantha* | Fabaceae | 17.6 | 500 |
| BCN06_1 | 0 | NA | NA | 26.1 | 430 |
| BCN06_2^b^ | 0 | *Geoffroea spinosa* | Fabaceae | 30 | 520 |
| BCN06_3^b^ | 0 | *Vachellia macracantha* | Fabaceae | 56 | 900 |
| BCN06_4^b^ | 0 | *Ipomoea* sp.1 | Convolvulaceae | 54.7 | 730 |
| BCN06_5^b^ | 0 | *Vachellia macracantha* | Fabaceae | 19.4 | 530 |
| BCN06_6^b^ | 0 | *Vachellia macracantha* | Fabaceae | 33.4 | 750 |
| BCN06_7^b^ | 0 | *Geoffroea spinosa* | Fabaceae | 27 | 415 |
| BCN06_8 | 0 | *Ficus obtusifolia* | Moraceae | NA | 1500 |
| BCN06_9 | 0 | *Ceiba insignis* | Malvaceae | NA | 2100 |
| BCN06_10 | 0 | *Ipomoea pauciflora* | Convolvulaceae | NA | 700 |
| BCN06_11^b^ | 0 | *Ipomoea pauciflora* | Convolvulaceae | 34.4 | 650 |
| BCN06_12 | 0 | NA | NA | 44.4 | 350 |
| BCN06_13^b^ | 0 | *Acnistus arborescens* | Solanaceae | 22 | 600 |
| BMN01_1^b^ | 0 | *Morus celtidifolia* | Moraceae | 58 | 1000 |
| BMN01_2^b^ | 0 | *Cedrela fissilis* | Meliaceae | 82.2 | 1200 |
| BMN01_3^b^ | 0 | *Trema micrantha* | Cannabaceae | 37 | 900 |
| BMN01_4 ^b^ | 0 | Undetermined sp.2 | NA | 97.5 | 520 |
| BMN01_6^b^ | 0 | *Cedrela fissilis* | Meliaceae | 73 | 750 |
| BMN01_7^b^ | 0 | *Cedrela fissilis* | Meliaceae | 67.6 | 700 |
| BMN01_8 ^b^ | 0 | Undetermined sp.3 | NA | 74.2 | 1400 |
| BMN01_9 ^b^ | 0 | Undetermined sp.2 | NA | 95.4 | 1200 |
| BMN01_10^b^ | 0 | *Vachellia macracantha* | Fabaceae | 68.4 | 700 |
| BMN02_1^b^ | 0 | *Leucaena trichodes* | Fabaceae | 160 | 2000 |
| BMN02_2^b^ | 0 | *Condalia weberbaueri* | Rhamnaceae | 150 | 1200 |
| BMN02_3 | 0 | NA | NA | 117 | 150 |
| BMN02_4 | 0 | NA | NA | 82.4 | NA |
| BMN02_6^b^ | 0 | *Ipomoea pauciflora* | Convolvulaceae | 53.4 | 600 |
| BMN02_7^b^ | 0 | *Pisonia aculeata* | Nyctaginaceae | 39 | 650 |
| BMN03_1^b^ | 0 | *Vachellia macracantha* | Fabaceae | 95 | 1000 |
| BMN03_2^b^ | 0 | *Leucaena trichodes* | Fabaceae | 121 | 1100 |
| BMN03_3^b^ | 0 | *Ceiba insignis* | Malvaceae | 549 | 1200 |
| BMN03_4^b^ | 0 | *Bursera graveolens* | Burseraceae | 124 | 800 |
| BMN03_5^b^ | 0 | *Vachellia macracantha* | Fabaceae | 106 | 1000 |
| BMN03_6 ^b^ | 0 | Undetermined sp.2 | NA | 190 | 1000 |
| BMN03_7^b^ | 0 | *Ceiba insignis* | Malvaceae | 159 | 1150 |
| BMN03_8^b^ | 0 | *Aegiphyla* cf. | Lamiaceae | 87 | 800 |
| BMN04_1 ^b^ | 0 | Undetermined sp.2 | NA | 304 | 1100 |
| BMN04_2^b^ | 0 | *Aegiphyla* cf. | Lamiaceae | 64 | 800 |
| BMN04_3^b^ | 0 | *Morus celtidifolia* | Moraceae | 50 | 1200 |
| BMN04_4^b^ | 0 | *Pisonia aculeata* | Nyctaginaceae | 63 | 1200 |
| BMN04_5^b^ | 0 | *Condalia weberbaueri* | Rhamnaceae | 132 | 1300 |
| BMN04_7^b^ | 0 | *Maytenus* cf. *retusa* | Celastraceae | 92 | 1000 |
| BMN04_8^b^ | 0 | *Senna mollissima* | Fabaceae | 35 | 1100 |
| BMN04_9^b^ | 0 | *Senna mollissima* | Fabaceae | 36.4 | 900 |
| BMN05_1^b^ | 0 | *Erythrina* sp. | Fabaceae | 72 | 800 |
| BMN05_2^b^ | 0 | *Bursera graveolens* | Burseraceae | 62 | 1300 |
| BMN05_3^b^ | 0 | *Aegiphyla* cf. | Lamiaceae | 52 | 530 |
| BMN05_4 ^b^ | 0 | Indetermined sp.1 | NA | 102 | 800 |
| BMN05_5^b^ | 0 | *Erythrina* sp. | Fabaceae | 105 | 1000 |
| BMN06_1^b^ | 0 | *Cedrela fissilis* | Meliaceae | 58 | 600 |
| BMN06_2^b^ | 0 | *Trema micrantha* | Cannabaceae | 100 | 1200 |
| BMN06_3^b^ | 0 | *Trema micrantha* | Cannabaceae | 96 | 1330 |
| BMN06_4^b^ | 0 | *Trema micrantha* | Cannabaceae | 55 | 1200 |
| BMN06_5^b^ | 0 | *Vachellia macracantha* | Fabaceae | 133 | 700 |
| BMN06_6^b^ | 0 | *Cedrela fissilis* | Meliaceae | 73 | 700 |
| BMN06_7 | 0 | *Erythrina* sp. | Fabaceae | NA | 1000 |
| BMN06_8^b^ | 0 | *Erythina* sp. | Fabaceae | 82 | 1100 |
| BMN06_9^b^ | 0 | *Trema micrantha* | Cannabaceae | 39 | 800 |
| BMN06_10^b^ | 0 | *Bursera graveolens* | Burseraceae | 33 | 900 |
| BMN06_11^b^ | 0 | *Bursera graveolens* | Burseraceae | 52 | 420 |
| BMN06_12^b^ | 0 | *Bursera graveolens* | Burseraceae | 50 | 800 |
| BMN06_13 | 0 | *Cedrela fissilis* | Meliaceae | 54.8 | NA |
| BMN07_1^b^ | 0 | *Eriotheca ruizii* | Malvaceae | 67.1 | 800 |
| BMN07_2^b^ | 0 | *Maytenus* cf. *retusa* | Celastraceae | 84 | 700 |
| BMN07_3 ^b^ | 0 | Indetermined sp.2 | NA | 444 | 1000 |
| BMN08_1^b^ | 0 | *Morus celtidifolia* | Moraceae | 39 | 600 |
| BMN08_2^b^ | 0 | *Eriotheca ruizii* | Malvaceae | 44 | 1200 |
| BMN08_3^b^ | 0 | *Morus celtidifolia* | Moraceae | 184 | 600 |
| BMN08_4^b^ | 0 | *Erythrina* sp. | Fabaceae | 105 | 1000 |
| BMN08_5 ^b^ | 0 | Undetermined sp.*2* | NA | 102 | 800 |
| BMN08_6^b^ | 0 | *Vachellia macracantha* | Fabaceae | 68 | 700 |
| BMN08_7^b^ | 0 | *Erythrina* sp. | Fabaceae | 52 | 550 |
| BMN08_8^b^ | 0 | *Erythrina* sp. | Fabaceae | 63 | 550 |
| BMN09_1 | 0 | *Acnistus arborescens* | Solanaceae | 29.2 | NA |
| BMN09_2 | 0 | *Acnistus arborescens* | Solanaceae | 30 | NA |
| BMN09_3 | 0 | *Acnistus arborescens* | Solanaceae | 24.3 | NA |
| BMN09_4 | 0 | *Acnistus arborescens* | Solanaceae | 42 | NA |
| BMN09_5 | 0 | *Cestrum auriculatum* | Solanaceae | 16 | NA |
| BMN09_6 | 0 | *Acnistus arborescens* | Solanaceae | 34.3 | NA |
| BMN09_7 | 0 | *Acnistus arborescens* | Solanaceae | 36 | NA |
| BMN09_8 | 0 | *Ipomoea pauciflora* | Convolvulaceae | 33 | NA |
| BMN09_9 | 0 | *Aegiphyla* cf. | Lamiaceae | 20.7 | NA |
| BMN09_10 | 0 | *Morus celtidifolia* | Moraceae | 46.5 | NA |
| BMN09_11 | 0 | *Acnistus arborescens* | Solanaceae | 23 | NA |
| BMN09_12 | 0 | *Ipomoea pauciflora* | Convolvulaceae | 69.4 | NA |
| BMN09_13 | 0 | *Ipomoea pauciflora* | Convolvulaceae | 31 | NA |
| BMN09_14 | 0 | *Acnistus arborescens* | Solanaceae | 30 | NA |
| BMN09_15 | 0 | *Ipomoea pauciflora* | Convolvulaceae | 63.2 | NA |
| BMN09_16 | 0 | *Ipomoea pauciflora* | Convolvulaceae | 76.2 | NA |
| BMN09_17 | 0 | *Ceiba insignis* | Malvaceae | 139.8 | NA |
| BMN09_18 | 0 | *Pisonia aculeata* | Nyctaginaceae | 59.3 | NA |
| BMN09_19 | 0 | *Trema micrantha* | Cannabaceae | 65.5 | NA |
| BMN09_20 | 0 | NA | NA | 93.8 | NA |
| BMN10_1 | 0 | *Cedrela fissilis* | Meliaceae | 75 | NA |
| BMN10_2 | 0 | *Pisonia aculeata* | Nyctaginaceae | 24.5 | NA |
| BMN10_3 | 0 | *Cedrela fissilis* | Meliaceae | 95 | NA |
| BMN10_4 | 0 | *Bursera graveolens* | Burseraceae | 53.3 | NA |
| BMN10_5 | 0 | *Fabaceae* sp.*1* | Fabaceae | 22 | NA |
| BMN10_6 | 0 | *Cedrela fissilis* | Meliaceae | 73 | NA |
| BMN10_7 | 0 | *Tecoma stans* | Bignoniaceae | 18.9 | NA |
| BMN10_8 | 0 | *Cedrela fissilis* | Meliaceae | 57.4 | NA |
| BMN10_9 | 0 | *Cedrela fissilis* | Meliaceae | 74.5 | NA |
| BMN10_10 | 0 | *Vachellia macracantha* | Fabaceae | 81.8 | NA |
| BMN10_11 | 0 | *Vachellia macracantha* | Fabaceae | 82 | NA |
| BMN10_12 | 0 | *Vachellia macracantha* | Fabaceae | 69 | NA |
| BMN10_13 | 0 | *Cedrela fissilis* | Meliaceae | 21.8 | NA |
| BMN10_14 | 0 | *Tecoma stans* | Bignoniaceae | 18.4 | NA |
| BMN10_15 | 0 | *Tecoma stans* | Bignoniaceae | 24 | NA |
| BMN10_16 | 0 | *Vachellia macracantha* | Fabaceae | 36.9 | NA |
| BMN10_17 | 0 | *Trema micrantha* | Cannabaceae | 61.6 | NA |
| BMN10_18 | 0 | *Vachellia macracantha* | Fabaceae | 33.3 | NA |
| BMN16_1 | 0 | *Eupatorium* sp. | Asteraceae | 26 | NA |
| BMN16_2 | 0 | *Pisonia aculeata* | Nyctaginaceae | 25 | NA |
| BMN16_3 | 0 | Undetermined sp.2 | NA | 250 | NA |
| BMN16_4 | 0 | *Cedrela fissilis* | Meliaceae | 49 | NA |
| BMN16_5 | 0 | *Aegiphyla* cf. | Lamiaceae | 65 | NA |
| BMN16_6 | 0 | *Pisonia aculeata* | Nyctaginaceae | 80 | NA |
| BMN16_7 | 0 | *Aegiphyla* cf. | Lamiaceae | 49 | NA |
| BMN16_8 | 0 | *Cedrela fissilis* | Meliaceae | 79 | NA |
| BMN16_9 | 0 | *Ipomoea pauciflora* | Convolvulaceae | 40 | NA |
| BMN16_10 | 0 | *Vachellia macracantha* | Fabaceae | 135 | NA |
| BMN16_11 | 0 | *Pisonia aculeata* | Nyctaginaceae | 106 | NA |
| BMN16_12 | 0 | *Acnistus arborescens* | Solanaceae | 21 | NA |
| BMN21_1 | 0 | *Acnistus arborescens* | Solanaceae | 29.5 | NA |
| BMN21_2 | 0 | *Acnistus arborescens* | Solanaceae | 12 | NA |
| BMN21_3 | 0 | *Acnistus arborescens* | Solanaceae | 19.5 | NA |
| BMN21_4 | 0 | *Ipomoea pauciflora* | Convolvulaceae | 37 | NA |
| BMN21_5 | 0 | *Eupatorium* sp. | Asteraceae | 24 | NA |
| BMN21_6 | 0 | *Senna mollissima* | Fabaceae | 40 | NA |
| BMN21_7 | 0 | *Cestrum auriculatum* | Solanaceae | 11 | NA |
| BMN21_8 | 0 | *Acnistus arborescens* | Solanaceae | 38 | NA |
| BMN21_9 | 0 | *Acnistus arborescens* | Solanaceae | 26 | NA |
| BMN21_10 | 0 | *Geoffroea spinosa* | Fabaceae | 76.5 | NA |
| BMN21_11 | 0 | *Pisonia aculeata* | Nyctaginaceae | 109 | NA |
| BMN21_12 | 0 | *Citharexylum* sp.*.1* | Verbenaceae | 90 | NA |
| BMN21_13 | 0 | *Acnistus arborescens* | Solanaceae | 17 | NA |
| BMN21_14 | 0 | *Pisonia aculeata* | Nyctaginaceae | 63 | NA |
| BMN21_15 | 0 | *Vachellia macracantha* | Fabaceae | 141 | NA |
| BMN24_1 | 0 | *Styrax* cf. *tomentosus* | Styracaceae | 73 | NA |
| BMN24_2 | 0 | *Eriotheca ruizii* | Malvaceae | 183 | NA |
| BMN24_3 | 0 | *Cedrela fissilis* | Meliaceae | 68.3 | NA |
| BMN24_4 | 0 | *Styrax* cf. *tomentosus* | Styracaceae | 24.8 | NA |
| BMN24_5 | 0 | *Cedrela fissilis* | Meliaceae | 31.5 | NA |
| BMN24_6 | 0 | *Senna mollissima* | Fabaceae | 22.5 | NA |
| BMN24_7 | 0 | *Cedrela fissilis* | Meliaceae | 63.2 | NA |
| BMN24_8 | 0 | *Senna mollissima* | Fabaceae | 12 | NA |
| BMN24_9 | 0 | *Pisonia aculeata* | Nyctaginaceae | 104.1 | NA |
| BMN24_10 | 0 | *Fabaceae* sp.*1* | Fabaceae | 36.5 | NA |
| BMN24_11 | 0 | *Cedrela fissilis* | Meliaceae | 52.6 | NA |
| BMN24_12 | 0 | *Cedrela fissilis* | Meliaceae | 40.2 | NA |
| BMN24_13 | 0 | *Pisonia aculeata* | Nyctaginaceae | 48.7 | NA |
| BMN24_14 | 0 | *Trema micrantha* | Cannabaceae | 91.8 | NA |
| BMN24_15 | 0 | *Eriotheca ruizii* | Malvaceae | 158.7 | NA |
| BMN24_16 | 0 | *Trema micrantha* | Cannabaceae | 41 | NA |
| BMN24_17 | 0 | *Ipomoea pauciflora* | Convolvulaceae | 38.5 | NA |
| BMN24_18 | 0 | *Ipomoea pauciflora* | Convolvulaceae | 15 | NA |
| BMN25_1 | 0 | *Acnistus arborescens* | Solanaceae | 31.3 | NA |
| BMN25_2 | 0 | *Fabaceae* sp.*1* | Fabaceae | 12.5 | NA |
| BMN25_3 | 0 | *Cedrela fissilis* | Meliaceae | 18.6 | NA |
| BMN25_4 | 0 | *Cestrum auriculatum* | Solanaceae | 18.2 | NA |
| BMN25_5 | 0 | *Cestrum auriculatum* | Solanaceae | 16 | NA |
| BMN25_6 | 0 | *Cestrum auriculatum* | Solanaceae | 32.7 | NA |
| BMN25_7 | 0 | *Chionanthus pubescens* | Oleaceae | 19.4 | NA |
| BMN25_8 | 0 | NA | NA | 311.7 | NA |
| BMN25_9 | 0 | *Cedrela fissilis* | Meliaceae | 24.5 | NA |
| BMN25_10 | 0 | *Ipomoea pauciflora* | Convolvulaceae | 55 | NA |
| BMN25_11 | 0 | *Ipomoea pauciflora* | Convolvulaceae | 64.5 | NA |
| BMN25_12 | 0 | *Pisonia aculeata* | Nyctaginaceae | 94 | NA |
| BMN25_13 | 0 | *Geoffroea spinosa* | Fabaceae | 18.6 | NA |
| BMN25_14 | 0 | *Cedrela fissilis* | Meliaceae | 51.9 | NA |
| BMN25_15 | 0 | *Ipomoea pauciflora* | Convolvulaceae | 90 | NA |
| BMN25_16 | 0 | *Bursera graveolens* | Burseraceae | 91 | NA |
| BMN26_1 | 0 | *Trema micrantha* | Cannabaceae | 216.6 | NA |
| BMN26_2 | 0 | *Cedrela fissilis* | Meliaceae | 86.8 | NA |
| BMN26_3 | 0 | *Cedrela fissilis* | Meliaceae | 31.4 | NA |
| BMN26_4 | 0 | *Senna incarnata* | Fabaceae | 83.2 | NA |
| BMN26_5 | 0 | *Vachellia macracantha* | Fabaceae | 141 | NA |
| BMN26_6 | 0 | *Cedrela fissilis* | Meliaceae | 83 | NA |
| BMN26_7 | 0 | *Vachellia macracantha* | Fabaceae | 82.8 | NA |
| BMN26_8 | 0 | *Pisonia aculeata* | Nyctaginaceae | 75.2 | NA |
| BMN26_9 | 0 | *Cedrela fissilis* | Meliaceae | 69.3 | NA |
| BMN26_10 | 0 | *Cedrela fissilis* | Meliaceae | 86 | NA |
| BMN26_11 | 0 | *Vachellia macracantha* | Fabaceae | 74 | NA |
| BMN26_12 | 0 | *Cedrela fissilis* | Meliaceae | 16 | NA |
| BMN26_13 | 0 | *Acnistus arborescens* | Solanaceae | 28.2 | NA |
| BMN27_1 | 0 | *Pisonia aculeata* | Nyctaginaceae | 87 | NA |
| BMN27_2 | 0 | *Pisonia aculeata* | Nyctaginaceae | 77.5 | NA |
| BMN27_3 | 0 | *Pisonia aculeata* | Nyctaginaceae | 124 | NA |
| BMN27_4 | 0 | *Vachellia macracantha* | Fabaceae | 111.4 | NA |
| BMN27_5 | 0 | *Acnistus arborescens* | Solanaceae | 26.4 | NA |
| BMN27_6 | 0 | *Pisonia aculeata* | Nyctaginaceae | 33 | NA |
| BMN27_7 | 0 | *Vachellia macracantha* | Fabaceae | 51.6 | NA |
| BMN27_8 | 0 | *Ipomoea pauciflora* | Convolvulaceae | 111 | NA |
| BMN27_9 | 0 | *Vachellia macracantha* | Fabaceae | 192.2 | NA |
| BMN27_10 | 0 | *Ipomoea pauciflora* | Convolvulaceae | 76.4 | NA |
| BMN27_11 | 0 | *Cedrela fissilis* | Meliaceae | 31.2 | NA |
| BMN27_12 | 0 | *Cedrela fissilis* | Meliaceae | 45.2 | NA |
| BMN27_13 | 0 | *Cedrela fissilis* | Meliaceae | 44 | NA |
| BMN31_1 | 0 | *Ceiba insignis* | Malvaceae | 328.4 | NA |
| BMN31_2 | 0 | *Ipomoea pauciflora* | Convolvulaceae | 50 | NA |
| BMN31_3 | 0 | *Tecoma stans* | Bignoniaceae | 15 | NA |
| BMN31_4 | 0 | *Vachellia macracantha* | Fabaceae | 67 | NA |
| BMN31_5 | 0 | *Bursera graveolens* | Burseraceae | 120.8 | NA |
| BMN31_6 | 0 | *Eriotheca ruizii* | Malvaceae | 92 | NA |
| BMN31_7 | 0 | *Eriotheca ruizii* | Malvaceae | 127 | NA |
| BMN31_8 | 0 | *Vachellia macracantha* | Fabaceae | 116.6 | NA |
| BMN31_9 | 0 | *Morus celtidifolia* | Moraceae | 43.4 | NA |
| BMN31_10 | 0 | *Bursera graveolens* | Burseraceae | 66 | NA |
| BMN32_1 | 0 | *Trema micrantha* | Cannabaceae | 13.9 | NA |
| BMN32_2 | 0 | *Acnistus arborescens* | Solanaceae | 12 | NA |
| BMN32_3 | 0 | *Morus celtidifolia* | Moraceae | 145 | NA |
| BMN32_4 | 0 | *Acnistus arborescens* | Solanaceae | 19 | NA |
| BMN32_5 | 0 | *Acnistus arborescens* | Solanaceae | 13.5 | NA |
| BMN32_6 | 0 | *Morus celtidifolia* | Moraceae | 101 | NA |
| BMN32_7 | 0 | Undetermined sp.4 | NA | 57 | NA |
| BMN32_8 | 0 | Undetermined sp.4 | NA | 68.8 | NA |
| BMN32_9 | 0 | *Ceiba insignis* | Malvaceae | 737 | NA |
| BMN32_10 | 0 | *Morus celtidifolia* | Moraceae | 34.5 | NA |
| BMN32_11 | 0 | *Ceiba insignis* | Malvaceae | 576 | NA |
| BMN32_12 | 0 | *Trema micrantha* | Cannabaceae | 82 | NA |
| BMN33_1 | 0 | *Senna mollissima* | Fabaceae | 26 | NA |
| BMN33_2 | 0 | *Vachellia macracantha* | Fabaceae | 73.8 | NA |
| BMN33_3 | 0 | *Bursera graveolens* | Burseraceae | 73 | NA |
| BMN33_4 | 0 | *Bursera graveolens* | Burseraceae | 74.7 | NA |
| BMN33_5 | 0 | *Vachellia macracantha* | Fabaceae | 102 | NA |
| CQN01_1^b^ | 0 | *Pisonia aculeata* | Nyctaginaceae | 74 | 700 |
| CQN01_2^b^ | 0 | *Vachellia macracantha* | Fabaceae | 61.6 | 700 |
| CQN01_3^b^ | 0 | *Vachellia macracantha* | Fabaceae | 106.3 | 800 |
| CQN01_4^b^ | 0 | *Vachellia macracantha* | Fabaceae | 86 | 850 |
| CQN01_5^b^ | 0 | *Pisonia aculeata* | Nyctaginaceae | 91.6 | 850 |
| CQN01_6^b^ | 0 | *Vachellia macracantha* | Fabaceae | 191 | 1000 |
| CQN01_7^b^ | 0 | *Pisonia aculeata* | Nyctaginaceae | 202 | 1100 |
| CQN01_8^b^ | 0 | *Vachellia macracantha* | Fabaceae | 159 | 850 |
| CQN02_1^b^ | 0 | *Vachellia macracantha* | Fabaceae | 30.4 | 900 |
| CQN02_2^b^ | 0 | *Vachellia macracantha* | Fabaceae | 60.9 | 950 |
| CQN02_3^b^ | 0 | *Vachellia macracantha* | Fabaceae | 32.5 | 700 |
| CQN02_4^b^ | 0 | *Vachellia macracantha* | Fabaceae | 80.4 | 750 |
| CQN02_5^b^ | 0 | *Vachellia macracantha* | Fabaceae | 25.6 | 750 |
| CQN02_6^b^ | 0 | *Vachellia macracantha* | Fabaceae | 31.6 | 600 |
| CQN02_7^b^ | 0 | *Vachellia macracantha* | Fabaceae | 15 | 400 |
| CQN02_8^b^ | 0 | *Vachellia macracantha* | Fabaceae | 17.3 | 450 |
| CQN02_9^b^ | 0 | *Vachellia macracantha* | Fabaceae | 26 | 450 |
| CQN02_10^b^ | 0 | *Vachellia macracantha* | Fabaceae | 18 | 480 |
| CQN02_11 | 0 | NA | NA | 31.6 | 800 |
| CQN02_13^b^ | 0 | *Vachellia macracantha* | Fabaceae | 23.8 | 400 |
| CQN02_15^b^ | 0 | *Vachellia macracantha* | Fabaceae | 60.9 | 750 |
| CQN02_16^b^ | 0 | *Vachellia macracantha* | Fabaceae | 34.5 | 650 |
| CQN02_17^b^ | 0 | *Vachellia macracantha* | Fabaceae | 16.7 | 500 |
| CQN02_18^b^ | 0 | *Vachellia macracantha* | Fabaceae | 30 | 530 |
| CQN02_19^b^ | 0 | *Vachellia macracantha* | Fabaceae | 63.8 | 800 |
| CQN03_1^b^ | 0 | *Pisonia aculeata* | Nyctaginaceae | 86 | 800 |
| CQN03_2^b^ | 0 | *Morus celtidifolia* | Moraceae | 61.9 | 700 |
| CQN03_3^b^ | 0 | *Pisonia aculeata* | Nyctaginaceae | 70 | 700 |
| CQN03_4 | 0 | NA | NA | 62 | 650 |
| CQN03_5^b^ | 0 | *Pisonia aculeata* | Nyctaginaceae | 55.9 | 700 |
| CQN03_6^b^ | 0 | *Vachellia macracantha* | Fabaceae | 59.5 | 650 |
| CQN03_7^b^ | 0 | *Vachellia macracantha* | Fabaceae | 69 | 700 |
| CQN03_8^b^ | 0 | *Vachellia macracantha* | Fabaceae | 41.8 | 650 |
| CQN03_9^b^ | 0 | *Pisonia aculeata* | Nyctaginaceae | 75.3 | 800 |
| CQN03_10^b^ | 0 | *Morus celtidifolia* | Moraceae | 91.2 | 850 |
| CQN04_1^b^ | 0 | *Pisonia aculeata* | Nyctaginaceae | 52.6 | 700 |
| CQN04_2^b^ | 0 | *Pisonia aculeata* | Nyctaginaceae | 56.4 | 700 |
| CQN04_3^b^ | 0 | *Pisonia aculeata* | Nyctaginaceae | 88.3 | 700 |
| CQN04_4^b^ | 0 | *Pisonia aculeata* | Nyctaginaceae | 100 | 650 |
| CQN04_5^b^ | 0 | *Acnistus arborescens* | Solanaceae | 25.2 | 550 |
| CQN04_6^b^ | 0 | *Pisonia aculeata* | Nyctaginaceae | 59.6 | 650 |
| CQN04_7^b^ | 0 | *Geoffroea spinosa* | Fabaceae | 65 | 700 |
| CQN04_8^b^ | 0 | *Pisonia aculeata* | Nyctaginaceae | 99 | 800 |
| CQN04_9^b^ | 0 | *Pisonia aculeata* | Nyctaginaceae | 50 | 1500 |
| CQN04_10^b^ | 0 | *Pisonia aculeata* | Nyctaginaceae | 126 | 750 |
| CQN04_11^b^ | 0 | *Pisonia aculeata* | Nyctaginaceae | 61.6 | 550 |
| CQN04_12^b^ | 0 | *Pisonia aculeata* | Nyctaginaceae | 120 | 550 |
| CQN05_1^b^ | 0 | *Vachellia macracantha* | Fabaceae | 84.3 | 800 |
| CQN05_2^b^ | 0 | *Pisonia aculeata* | Nyctaginaceae | 51.7 | 600 |
| CQN05_3^b^ | 0 | *Vachellia macracantha* | Fabaceae | 62 | 870 |
| CQN05_4^b^ | 0 | *Pisonia aculeata* | Nyctaginaceae | 85 | 1000 |
| CQN05_5^b^ | 0 | *Pisonia aculeata* | Nyctaginaceae | 88 | 1200 |
| CQN05_6^b^ | 0 | *Morus celtidifolia* | Moraceae | 89 | 830 |
| CQN05_7^b^ | 0 | *Zanthoxylum fagara* | Rutaceae | 25.9 | 500 |
| CQN05_8^b^ | 0 | *Pisonia aculeata* | Nyctaginaceae | 76.7 | 860 |
| CQN05_9^b^ | 0 | *Pisonia aculeata* | Nyctaginaceae | 230 | 1100 |
| CQN05_10^b^ | 0 | *Senna mollissima* | Fabaceae | 34 | 600 |
| CQN06_1^b^ | 0 | *Pisonia aculeata* | Nyctaginaceae | 112 | 1000 |
| CQN06_2^b^ | 0 | *Pisonia aculeata* | Nyctaginaceae | 52.2 | 900 |
| CQN06_3 | 0 | NA | NA | 144 | 1200 |
| CQN06_4^b^ | 0 | *Morus celtidifolia* | Moraceae | 65 | 1100 |
| CQN06_5^b^ | 0 | *Pisonia aculeata* | Nyctaginaceae | 45 | 550 |
| CQN06_6^b^ | 0 | *Pisonia aculeata* | Nyctaginaceae | 164 | 900 |
| CQN06_7^b^ | 0 | *Pisonia aculeata* | Nyctaginaceae | 90 | 1100 |
| CQN06_8 | 0 | NA | NA | 61.2 | 330 |
| CQN06_9^b^ | 0 | *Pisonia aculeata* | Nyctaginaceae | 110 | 700 |
| CQN06_10^b^ | 0 | *Pisonia aculeata* | Nyctaginaceae | 103 | 760 |
| CQN06_11 | 0 | NA | NA | 67.8 | 700 |
| CQN06_12 | 0 | NA | NA | 245 | 1300 |
| CQN07_1^b^ | 0 | *Vachellia macracantha* | Fabaceae | 77.9 | 1200 |
| CQN07_2^b^ | 0 | *Pisonia aculeata* | Nyctaginaceae | 127.6 | 1100 |
| CQN07_3^b^ | 0 | *Vachellia macracantha* | Fabaceae | 84 | 900 |
| CQN07_4^b^ | 0 | *Vachellia macracantha* | Fabaceae | 59.6 | 900 |
| CQN07_5 | 0 | NA | NA | 51.8 | 830 |
| CQN07_6^b^ | 0 | *Vachellia macracantha* | Fabaceae | 52.7 | 770 |
| CQN07_7^b^ | 0 | *Vachellia macracantha* | Fabaceae | 18.6 | 900 |
| CQN07_8^b^ | 0 | *Vachellia macracantha* | Fabaceae | 56 | 700 |
| CQN07_9^b^ | 0 | *Vachellia macracantha* | Fabaceae | 69.5 | 1100 |
| CQN07_10^b^ | 0 | *Zanthoxylum fagara* | Rutaceae | 21.4 | 708 |
| CQN07_11^b^ | 0 | *Pisonia aculeata* | Nyctaginaceae | 268 | 1300 |
| CQN07_12^b^ | 0 | *Vachellia macracantha* | Fabaceae | 31.6 | 600 |
| CQN08_1^b^ | 0 | *Pisonia aculeata* | Nyctaginaceae | 66.9 | 900 |
| CQN08_2^b^ | 0 | *Pisonia aculeata* | Nyctaginaceae | 64.2 | 900 |
| CQN08_3^b^ | 0 | *Pisonia aculeata* | Nyctaginaceae | 220 | 1280 |
| CQN08_4^b^ | 0 | *Vachellia macracantha* | Fabaceae | 58.3 | 1160 |
| CQN08_5^b^ | 0 | *Vachellia macracantha* | Fabaceae | 86 | 1200 |
| CQN08_6 | 0 | NA | NA | 51.8 | 830 |
| CQN08_7^b^ | 0 | *Vachellia macracantha* | Fabaceae | 56 | 700 |
| CQN09_1^b^ | 0 | *Vachellia macracantha* | Fabaceae | 77.7 | 1000 |
| CQN09_2^b^ | 0 | *Vachellia macracantha* | Fabaceae | 63.6 | 1400 |
| CQN09_3^b^ | 0 | *Pisonia aculeata* | Nyctaginaceae | 105 | 1300 |
| CQN09_4^b^ | 0 | *Pisonia aculeata* | Nyctaginaceae | 99.2 | 1000 |
| CQN09_5 | 0 | NA | NA | 82.7 | 1250 |
| CQN09_6^b^ | 0 | *Morus celtidifolia* | Moraceae | 124.6 | 1400 |
| CQN09_7^b^ | 0 | *Vachellia macracantha* | Fabaceae | 50 | 900 |
| CQN09_8^b^ | 0 | *Vachellia macracantha* | Fabaceae | 129.6 | 1300 |
| CQN09_9 | 0 | NA | NA | 121 | 1350 |
| CQN10_1^b^ | 0 | *Pisonia aculeata* | Nyctaginaceae | 77.8 | 800 |
| CQN10_2^b^ | 0 | *Pisonia aculeata* | Nyctaginaceae | 52.2 | 969 |
| CQN10_3^b^ | 0 | *Morus celtidifolia* | Moraceae | 66.8 | 1200 |
| CQN10_4^b^ | 0 | *Pisonia aculeata* | Nyctaginaceae | 80 | 800 |
| CQN10_5^b^ | 0 | *Vachellia macracantha* | Fabaceae | 34.3 | 500 |
| CQN10_6^b^ | 0 | *Vachellia macracantha* | Fabaceae | 48 | 730 |
| CQN10_7^b^ | 0 | *Vachellia macracantha* | Fabaceae | 50 | 700 |
| CQN10_8^b^ | 0 | *Vachellia macracantha* | Fabaceae | 89.5 | 1100 |
| CQN10_9^b^ | 0 | *Acnistus arborescens* | Solanaceae | 24.6 | 500 |
| CQN10_10^b^ | 0 | *Morus celtidifolia* | Moraceae | 55.8 | 1200 |
| ^a^  0 = absence of nest, 1 = presence of nest; ^b^ Samples used for predictors of nesting-habitat preferences; NA = no data. | | | | | |
