# Supplementary material for: Influence of ecological factors on the presence of a triatomine species associated with the arboreal habitat of a host of Trypanosoma cruzi
Source: Parasit Vectors. 2018 Oct 29;11:567. doi: 10.1186/s13071-018-3138-4 (PMC6206927; doi:10.1186/s13071-018-3138-4)
Supplement: Supplementary file 2 — Table S2. Effect on nest-habitat features. Multi-model inference data used to evaluate the effects on triatomine abundance, colonization and their infection with T. cruzi. (DOCX 18 kb) [file 13071_2018_3138_MOESM2_ESM.docx]

**Additional file 2**

**Table S2.** Effect of nest-habitat features. Multi-model inference data used to evaluate the effects on triatomine abundance, colonization and their infection with *T. cruzi*.

|  | **Nest Code** | **TA** | **TR** | **NH (cm)** | **TH (cm)** | **NO^a^** | **ABUND** | **COL^b^** | **INF^c^** |
| --- | --- | --- | --- | --- | --- | --- | --- | --- | --- |
| 1 | BCN01^d, e^ | 6 | 5 | 870 | 964 | 0 | 1 | 1 | 2 |
| 2 | BCN02^d, e^ | 22 | 4 | 1049 | 1089 | 1 | 5 | 2 | 2 |
| 3 | BCN03^d, e^ | 30 | 4 | 970 | 1255 | 1 | 3 | 2 | 2 |
| 4 | BCN04^d, e^ | 42 | 8 | 1365 | 2000 | 1 | 1 | 1 | 1 |
| 5 | BCN05^d, e^ | 16 | 6 | 1061 | 1462 | 1 | 7 | 2 | 2 |
| 6 | BCN06^d^ | 13 | 10 | 540 | 651 | 0 | 0 | 0 | 0 |
| 7 | BMN01^d, e^ | 9 | 7 | 1000 | 1200 | 0 | 9 | 2 | 2 |
| 8 | BMN02^d^ | 6 | 7 | 2579 | 2653 | 1 | 0 | 0 | 0 |
| 9 | BMN03^d^ | 8 | 6 | 816 | 997 | 0 | 0 | 0 | 0 |
| 10 | BMN04 | 8 | 7 | NA | 1600 | 0 | 0 | 0 | 0 |
| 11 | BMN05^d, e^ | 5 | 5 | 700 | 850 | 0 | 10 | 2 | 1 |
| 12 | BMN06^d, e^ | 13 | 5 | 893 | 900 | 1 | 1 | 1 | 1 |
| 13 | BMN07^d, e^ | 3 | 4 | 1000 | 1400 | 0 | 2 | 2 | 2 |
| 14 | BMN08^d^ | 8 | 5 | 650 | 800 | 1 | 0 | 0 | 0 |
| 15 | BMN09 | 20 | 9 | NA | 1389 | 1 | 0 | 0 | 0 |
| 16 | BMN10 | 18 | 7 | NA | 1161 | NA | 0 | 0 | 0 |
| 17 | BMN11 | NA | NA | 1250 | 1400 | NA | NA | NA | NA |
| 18 | BMN12 | NA | NA | 1250 | 1400 | 0 | 1 | 1 | 1 |
| 19 | BMN13 | NA | NA | 1300 | 1400 | NA | NA | NA | NA |
| 20 | BMN14 | NA | NA | 1264 | 1439 | NA | NA | NA | NA |
| 21 | BMN15 | NA | NA | 1114 | 1439 | NA | NA | NA | NA |
| 22 | BMN16^d, e^ | 12 | 8 | 714 | 1264 | 1 | 2 | 1 | 1 |
| 23 | BMN17 | NA | NA | 1434 | 2964 | NA | NA | NA | NA |
| 24 | BMN18 | NA | NA | 1104 | 1764 | NA | NA | NA | NA |
| 25 | BMN19 | NA | NA | 639 | 2964 | NA | NA | NA | NA |
| 26 | BMN20 | NA | NA | 1864 | 2964 | NA | NA | NA | NA |
| 27 | BMN21^d, e^ | 15 | 9 | 1164 | 2864 | 1 | 26 | 2 | 2 |
| 28 | BMN22 | NA | NA | 1164 | 1764 | NA | NA | NA | NA |
| 29 | BMN23 | NA | NA | 1114 | 1564 | NA | NA | NA | NA |
| 30 | BMN24^d, e^ | 18 | 8 | 964 | 1114 | 1 | 55 | 2 | 2 |
| 31 | BMN25^d, e^ | 16 | 10 | 764 | 864 | 1 | 1 | 1 | 2 |
| 32 | BMN26^d, e^ | 13 | 6 | 914 | 1164 | 0 | 60 | 2 | 2 |
| 33 | BMN27^d, e^ | 13 | 5 | 925 | 1274 | 0 | 32 | 2 | 2 |
| 34 | BMN28 | NA | NA | NA | NA | 0 | 0 | 0 | 0 |
| 35 | BMN29 | NA | NA | NA | NA | 1 | 2 | 1 | 2 |
| 36 | BMN30 | NA | NA | NA | NA | 0 | 2 | 1 | 2 |
| 37 | BMN31^d^ | 10 | 7 | 764 | 1064 | 1 | 0 | 0 | 0 |
| 38 | BMN32^d^ | 12 | 5 | 1214 | 1614 | 0 | 0 | 0 | 0 |
| 39 | BMN33^d^ | 5 | 3 | 1014 | 1064 | 0 | 0 | 0 | 0 |
| 40 | BMNR01^d, e^ | 9 | 7 | 1000 | 1200 | 1 | 21 | 2 | 2 |
| 41 | BMNR09^d, e^ | 20 | 9 | 1189 | 1389 | 0 | 24 | 2 | 2 |
| 42 | BMNR10 | 18 | 7 | 639 | 1161 | NA | 0 | 0 | 0 |
| 43 | CQN01 | 8 | 2 | 437 | 655 | 0 | 14 | 2 | NA |
| 44 | CQN02^d^ | 17 | 2 | 1074 | 1370 | 0 | 0 | 0 | 0 |
| 45 | CQN03^d^ | 10 | 4 | 590 | 1510 | 0 | 0 | 0 | 0 |
| 46 | CQN04^d, e^ | 12 | 3 | 702 | 960 | 0 | 2 | 1 | 2 |
| 47 | CQN05 | 10 | 5 | NA | 830 | 1 | 2 | 1 | 2 |
| 48 | CQN06^d^ | 12 | 6 | 917 | 1100 | 1 | 0 | 0 | 0 |
| 49 | CQN07^d^ | 12 | 4 | 606 | 1048 | 1 | 0 | 0 | 0 |
| 50 | CQN08^d, e^ | 7 | 4 | 757 | 900 | 0 | 8 | 2 | 2 |
| 51 | CQN09 | 9 | 5 | NA | 1180 | 0 | 0 | 0 | 0 |
| 52 | CQN10^d, e^ | 10 | 4 | 600 | 1100 | 1 | 7 | 2 | 2 |
| ^a^ 0 = abandoned nest, 1= occupied nest; ^b^ 0 = non infested; 1 = infested without nymphs: 2 = infested with nymphs; ^c^ 0 = non infested with triatomines; 1 = non infected with *T. cruzi*; 2 = infected with *T. cruzi*; ^d^ Samples used for the analysis of predictors of triatomine abundance; ^e^ Samples used for the analysis of predictors of triatomine colonization an infection with trypanosomes; NA: no data | | | | | | | | | |
